# Indirect estimation of the need for palliative care during the COVID-19 pandemic: A descriptive cross-sectional study using mortality data in the Biobío Region, Chile

**DOI:** 10.1371/journal.pone.0288020

**Published:** 2023-07-07

**Authors:** Claudia Barría-Sandoval, Maritza Espinoza Venegas, Guillermo Ferreira

**Affiliations:** 1 Facultad de Ciencias para el Cuidado de la Salud, Universidad San Sebastián, Sede Concepción, Chile; 2 Faculty of Nursing, Universidad de Concepción, Concepción, Chile; 3 Department of Statistics, Universidad de Concepción, Concepción, Chile; 4 Research Nucleus on Comprehensive Community Care and Health Education (NICCES), Faculty of Health Sciences, Universidad de las Américas, Providencia, Chile; Universidade Federal do Rio Grande do Norte, BRAZIL

## Abstract

**Background:**

People with chronic diseases in their advanced phase require palliative care. This is essential to ensure their quality of life as it ends. However, a very low percentage of patients receive the necessary palliative care. The COVID-19 pandemic has adversely affected the planning and provision of palliative care. Despite this, in Chile, palliative care coverage was extended by law to cover nononcological chronic diseases. Implementation of this law is expected to be a significant challenge in terms of material resources, as well as the need for the formation of specialized palliative care teams. Therefore, it is essential to estimate the need for palliative care for all chronic diseases to generate useful input for planning and decision-making in public health.

**Objectives:**

To indirectly estimate the need for palliative care among people with Chronic Oncological Diseases (COD) and Chronic Non-Oncological Diseases (CNOD) during the prepandemic and pandemic context due to COVID-19 in the Biobío Region in Chile.

**Methods:**

Cross-sectional study based on mortality data from chronic oncological and nononcological diseases during the prepandemic (2010-2018) and pandemic (2020-2021) contexts due to COVID-19 in a Region of Chile through indirect estimation using minimal estimate, standardized mortality rates and geographically weighted regression.

**Results:**

It was estimated that 76.25% of deaths from chronic diseases in the Biobío Region would have required palliative care, which represents 77,618 people who should have been included in these health benefits. The pandemic had a significant effect on the average number of deaths from CNOD. People belonging to this group were more likely to die from COVID-19 than from their baseline disease, unlike the deaths of people from COD, where no significant changes were observed.

**Conclusion:**

These estimates highlight the potential size of the population requiring palliative care and emphasize the importance of recognizing the rights of individuals with COD and CNOD conditions. It is evident that there is a significant demand for palliative care services, as well as a pressing need for adequate resources, effective management, and strategic planning to cater to the needs of this population. This is particularly crucial in the heavily impacted areas and communes of the Biobío Region, Chile.

## Introduction

People with advanced chronic illnesses require palliative care, which is the active, holistic care of people of all ages with serious health-related suffering due to severe illness, especially near the end of life [1]. Palliative care is essential to ensure the quality of life at the end of treatment. However, only 14% of the world population receives the necessary palliative care, and by 2060, a global increase of 87% in serious health-related suffering amenable to receiving palliative care is projected [2].

Chile is in a stage of preliminary integration of standard palliative care health services [3]. The development of palliative care in the national context began in the 1980s; then, in 1994, the National Cancer Pain Relief and Palliative Care Program of the Chilean Ministry of Health was created [4]. In 2004, palliative care was incorporated into the Regime of Explicit Health Guarantees (GES) through Law 19,966 to grant access, coverage and financial protection to all people with chronic oncological disease (COD), excluding chronic nononcological diseases (CNOD) [5]. Recently, in 2022, Law 21,375 extended palliative care to all people suffering from terminal or serious illnesses, thus incorporating all chronic illnesses [6].

On the other hand, during recent decades in Chile, accelerated aging of the population has been observed. Between 1950 and 2015, the life expectancy of Chileans increased by 25 years, and in less than three decades, the group of people over 60 years of age went from being 10% of the total population of the country to 20%. This change in the population pyramid has led to a progressive increase in chronic diseases, which has projected higher palliative care requirements, especially in advanced stages of the disease and at the end of life [7]. The latest epidemiological data in Chile indicate that in 2019, 69% of deaths were concentrated in four groups of chronic diseases: circulatory (27.1%), malignant tumors (25%), respiratory (9.5%) and other external causes of mortality (7.5%) [7]. Similarly, the 2016–2017 National Health Survey showed that 78.5% of people over 15 years of age live with 2 or more simultaneous chronic diseases that require treatment at least once or twice a year for the rest of their life [8]. Considering these statistics, a great need for palliative care is inferred for this group of people at the national level, and its implementation for the entire group of chronic diseases is expected to be a significant challenge in terms of material resources as well as the formation of specialized palliative care teams. On the other hand, at the national and regional levels, there is a lack of estimates of the need for palliative care in patients with CNOD. Therefore, it is imperative to understand the real magnitude of the need for palliative care to generate guidance for the planning of public health policies.

Different studies have shown that it is possible to estimate the need for palliative care through indirect methods from death records [9–11]. These authors have used mortality registries to calculate age- and sex-specific proportions of deaths from chronic diseases to estimate the prevalence of the need for palliative care in the population in a given period. Likewise, they proposed geographical maps to locate the areas with the highest mortality rate from chronic diseases. This indirect measurement through mortality data can be very useful to understand the real epidemiology of chronic diseases and thus determine an approximation of the corresponding palliative care needs in people with COD and CNOD.

It is currently impossible to ignore the global context of the COVID-19 pandemic, which, as indicated in recent research [12–14], has shown the fragile nature of health systems for responding to pandemic threats as well as threats arising from chronic noncommunicable diseases, which increase the risks of severe disease and death from COVID-19. These studies consider that focusing solely on COVID-19 care is an error because the impact of the pandemic is due to both the action of the virus and its interaction with chronic diseases and weak or fragmented health care systems. Therefore, it is relevant to highlight and understand the need for palliative care by incorporating the pandemic context due to COVID-19 in at least one region of the country to plan and implement public health care strategies in a timely manner. In this sense, it is important to know the evolutionary dynamics of deaths from chronic diseases in the pre- and postpandemic periods, as well as to find the magnitude of the impact of COVID-19 on these diseases. Several authors have shown that COVID-19 has caused a significant decrease in some chronic diseases; for example, [15] pointed out that the variations between mortality rates in the pre- and postpandemic periods could be explained by a greater susceptibility to COVID-19 in patients with underlying chronic diseases, and [16] revealed a decrease in the number of deaths from respiratory diseases in all regions of Chile. These authors concluded that a possible cause of the decrease in mortality due to chronic diseases during the COVID-19 pandemic could be because of how each country defines the main cause of mortality. This would consequently lead to errors in the number of true causes of death in databases, which may have resulted in an overestimation of the mortality rate from COVID-19 and an underestimation of the mortality rate from other underlying chronic conditions. This study sought to determine the trend of deaths from chronic diseases in a region of Chile in pre- and post-COVID-19 pandemic periods and to estimate the population that would potentially require palliative care from death records in the Biobío Region, contributing to the general knowledge of the regional and, indirectly, the national reality regarding the need for palliative care among patients with COD and CNODs.

Finally, it should be noted that palliative care programs have been delayed by the COVID-19 pandemic, which implies that millions of people worldwide are affected by receiving palliative care in person and in a timely manner. [17] noted that approximately 13 million Americans require palliative care at the end of life, and [18] showed that, in Chile, there was a delay in palliative care due to the pandemic, where the priority was care and delivery of resources to people with COVID-19. This study aims to demonstrate the importance of responding to the new challenges brought by the pandemic, including the increase in the rates of people with chronic diseases. In light of the aforementioned, the hypothesis for this research is as follows: the demand for palliative care is greater among individuals with CNODs in comparison to those with COD throughout both the pre-pandemic and pandemic periods.

## Methods

### Study design and population

In order to estimate the potential demand for palliative care in the Biobío region, a descriptive cross-sectional analysis was conducted utilizing death registration data. The study included a population of 101,791 recorded of people who died from all causes and of all ages during the pre-pandemic period (2010–2018) and pandemic period (2020–2021) in 33 communes of the Biobío Region, Chile. Mortality records for 2019 were excluded from the analysis because they were not available.

### Data sources and definition of variables

**Fig 1:**
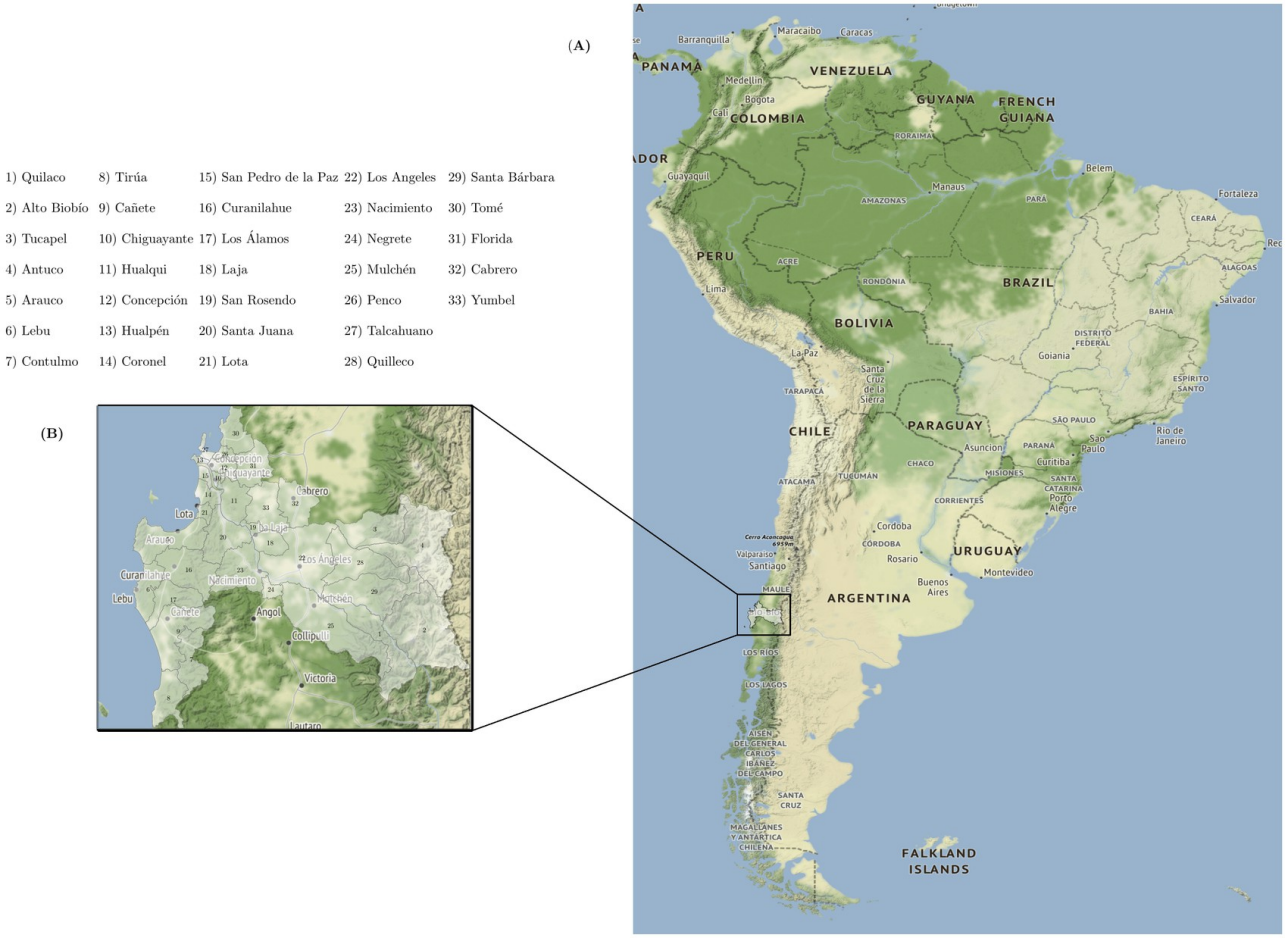
Geographical plot of the spatial distribution of the Biobío Region, Chile. **(A)** Geographical plot of Chile in South America. **(B)** Location of the 33 communes selected in the Biobío Region, Chile. Base map and data from OpenStreetMap and OpenStreetMap Foundation under the Open Database License.

Chilean mortality records were used, which are free and open access on the website of the Department of Statistics and Health Information (DEIS) of the Ministry of Health of Chile [19]. The selected and analyzed dataset provides information on age, sex, cause of death and place of death. Fig 1 displays the spatial distribution of the communes of the Biobío Region in southern Chile.

To determine the true epidemiology of chronic diseases and thus determine an approximation of the corresponding palliative care needs in people with CNOD, this study considered the proposal by [11], who used the ICD-10 classification (International Statistical Classification of Diseases and Related Health Problems-10th Revision). In this study, based on the proposal by Calvache et al.’s article, the following ICD-10 codes related to human immunodeficiency virus infection and acquired immunodeficiency syndrome (HIV/AIDS) were excluded: B20 and B23. These codes were excluded because they have their origin in infectious, parasitic diseases and other conditions that do not correspond to the group of diseases relevant to this study. This research focuses on chronic diseases that are not caused by infections, parasites or other conditions. Rather, the diseases have a progressive, gradual course, with varying degrees of impairment of autonomy and quality of life, which can contribute to medium- or even short-term death. Table 1 shows a description of the causes of death and the ICD-10 classification codes used to indicate mortality in the population that would have required palliative care. Deaths attributed to COD were determined based on the ICD-10 classification, specifically codes C00-C97, which encompass malignant neoplasms. A total of 26,635 deaths were identified within this category. Conversely, deaths related to CNOD were classified using the remaining codes of the ICD-10 classification, resulting in a total of 50,983 deaths.

**Table 1:** ICD-10 Codes for conditions identified as potentially having palliative care needs.

Underlying cause of death	ICD-10 codes
Malignant neoplasm	C00-C97
Heart & cerebrovascular disease	I00–I52 (excluding I12 and I13.1), I60–I69
Renal disease	N17, N18, N28, I12, I13.1
Liver disease	K70-K77
Respiratory disease	J06–J18, J20–J22, J40–J47, J96
Neurodegenerative disease	G10, G20, G35, G122, G903, G231
Alzheimer’s, dementia and senility	F01, F03, G30, R54
HIV/AIDS	B21, B22, B24

### Statistical analysis

Three methodological approaches were considered: minimum estimation, standardized mortality rates, and geographically weighted regression models, which are described below. Additionally, to detect significant differences between the number of deaths for each location in prepandemic and pandemic periods, we applied Welch’s t-statistic, which is defined at the end of this section.

#### Minimal estimate

The “minimal estimate” [9, 11] considers 10 diseases (Table 1) that may potentially require palliative care, such as cancer, heart failure, kidney failure, liver failure, chronic obstructive pulmonary disease, amyotrophic/motor neuron disease, lateral sclerosis, Parkinson’s disease, Huntington’s disease, Alzheimer’s disease, and HIV/AIDS. In this study, to determine the number of people potentially requiring palliative care in the Biobío Region, the minimal estimate approach was applied, calculating the age-and sex-specific proportions of deaths from the chronic progressive diseases defined in Table 1.

#### Standardized mortality rates

With this approach, a geographic analysis was carried out on the standardized mortality rates in the Biobío region between 2010 and 2021, omitting 2019 since no information was found on deaths for that year. This methodology was based on [11], who estimated the need for palliative care in a geographic study on mortality in Colombia between 2012 and 2016, calculating the crude rate per year in a population density graph. However, the levels of health and mortality rates between the regions and communes of Chile are not homogeneous, manifesting in particular in the heterogeneity in the age groups of the different communes considered in this study.

Due to the aforementioned factors, age-standardized death rates (ASDR) obtained by the direct method were used. In this regard, what was stated in the literature on ASDR was considered in this study to study the geographical behavior of the causes of mortality; for example, [20] used ASDR to examine aggregate divergence in mortality among southern US states, and [21, 22] investigated temporal trends through ASDR of deaths from all causes and from the leading causes of death in the US. Other investigations with this same approach were carried out by the authors cited in the references below [23, 24].

#### Geographically weighted regression (GWR)

To determine the effect of the COVID-19 pandemic on deaths from COD and CNOD that could have required palliative care, the average ASDR in the pandemic period (2020–2021) was used to formulate a linear relationship with the average mortality from COVID-19 and the historical average of ASDR in the prepandemic period (2010–2018). After that, the geographically weighted regression (GWR) method was applied ([25]), which allows the regression coefficients to depend on covariates such as the longitude and latitude of the communes of the Biobío Region [26]. In the GWR model, the classical or global regression coefficients are replaced by local parameters, as described in Eq (1).

$$\begin{array}{c} {\bar{ASDR}}_{i}=\beta_{0}\left(u_{i},v_{i}\right)+\beta_{1}\left(u_{i},v_{i}\right){\bar{ASDR}}_{i}^{*}+\beta_{2}\left(u_{i},v_{i}\right){\bar{COVID}}_{i}^{*}+\varepsilon_{i},\;\;i=1,2,\ldots ,n, \end{array}$$ (1)

where:

-
$\bar{ASDR}\left(i\right)$ is the average mortality rate standardized by age in commune *i* between 2020 and 2021
- (*u*_*i*_, *v*_*i*_) denotes the longitude and latitude coordinates of the *i*–th commune.
-
${\bar{ASDR}}^{*}\left(i\right)$ is the average historical standardized mortality rate by age in commune *i* for the period 2010–2018.
-
${\bar{COVID}}^{*}\left(i\right)$ represents the COVID-19 average death rate from 2020–2021.
- *ε* is the error.

The coefficient function vector ***β***(*u_i_*, *v_i_*) for the *i*–th observation in GWR can be estimated via the locally weighted least square procedure, which is available in the free R statistical software [27], through the GWmodel package developed by the authors in reference [28].

#### Welch’s t-statistic

To determine the trends in the number of deaths from COD and CNOD in all communes registered before and after COVID-19, the mean-variance tests before and after were considered, where the data from 2020 to 2021 corresponded to the pandemic period and 2010 to 2018 were from before the pandemic. To differentiate cases of nonhomogeneity of variance, the Welch ([29]) test was used in this study. The most important difference between Student’s t-test and Welch’s t-test is that, when both the variances and the sample size differ between groups, the t-value, degrees of freedom, and p-value all differ between Student’s t-test and Welch’s t-test. Welch’s t-statistic is defined as follows:

$$\begin{array}{c} t_{welch}=\frac{{\bar{X}}_{1}-{\bar{X}}_{2}}{\sqrt{\frac{s_{1}^{2}}{n_{1}}+\frac{s_{2}^{2}}{n_{2}}}}, \end{array}$$

where ${\bar{X}}_{j}=\frac{1}{n_{j}}\sum_{i=1}^{n_{j}}X_{ij}$ and $s_{j}^{2}=\frac{1}{n_{j}-1}\sum_{i=1}^{n_{j}}{\left(X_{ij}-{\bar{X}}_{j}\right)}^{2}$ represent the sample mean of the *j*–th population (*j* = 1, 2) and sample variance, respectively. As indicated by [30], the exact distribution of Welch’s t-statistic can be approximated, under the assumption of normality, by a t-distribution with degrees of freedom

$$\begin{array}{c} df=\frac{{\left(\frac{s_{1}^{2}}{n_{1}}+\frac{s_{2}^{2}}{n_{2}}\right)}^{2}}{\frac{{\left(\frac{s_{1}^{2}}{n_{1}}\right)}^{2}}{n_{1}-1}+\frac{{\left(\frac{s_{2}^{2}}{n_{2}}\right)}^{2}}{n_{2}-1}}. \end{array}$$

When both variances and sample sizes are the same in each independent group, the t-values, degrees of freedom, and p-values in Student’s t-test and Welch’s t-test are the same. Regarding the assumption of normality, several authors ([31, 32]) have studied the power of the Welch test and have agreed that the sampling distribution of means is close to being a normal distribution when means are based on sample sizes greater than 40. R software [27] (version 4.1.2, R 4.1.2: R Core Team. R: A Language and Environment for Statistical Computing. Vienna, Austria: R Foundation for Statistical Computing; 2019. Available online at: https://www.R-project.org/) was used for data analysis and graphs.

## Results

**Fig 2:**
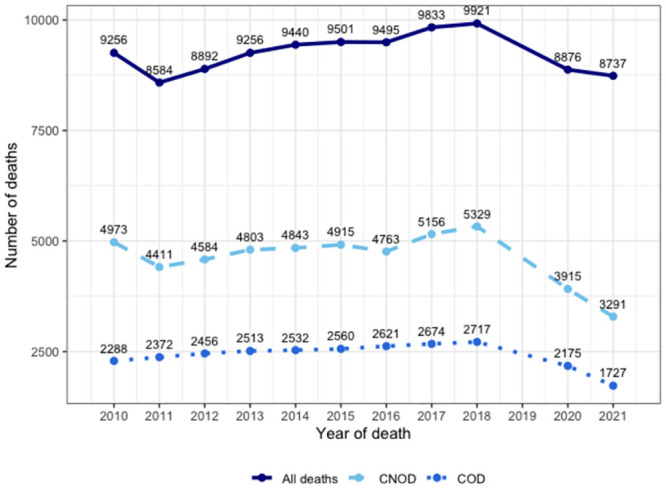
Prevalence of mortality and need for palliative care. Absolute values of deaths from any cause and number of deaths that potentially required palliative care from 2010 to 2021 in the Biobío Region, Chile. Solid lines indicate prevalence of deaths from chronic oncological disease (COD) and chronic nononcological diseases (CNOD). Dashed lines indicate the prevalence of deaths from CNOD. The dotted line represents the prevalence deaths due to COD.

**Fig 3:**
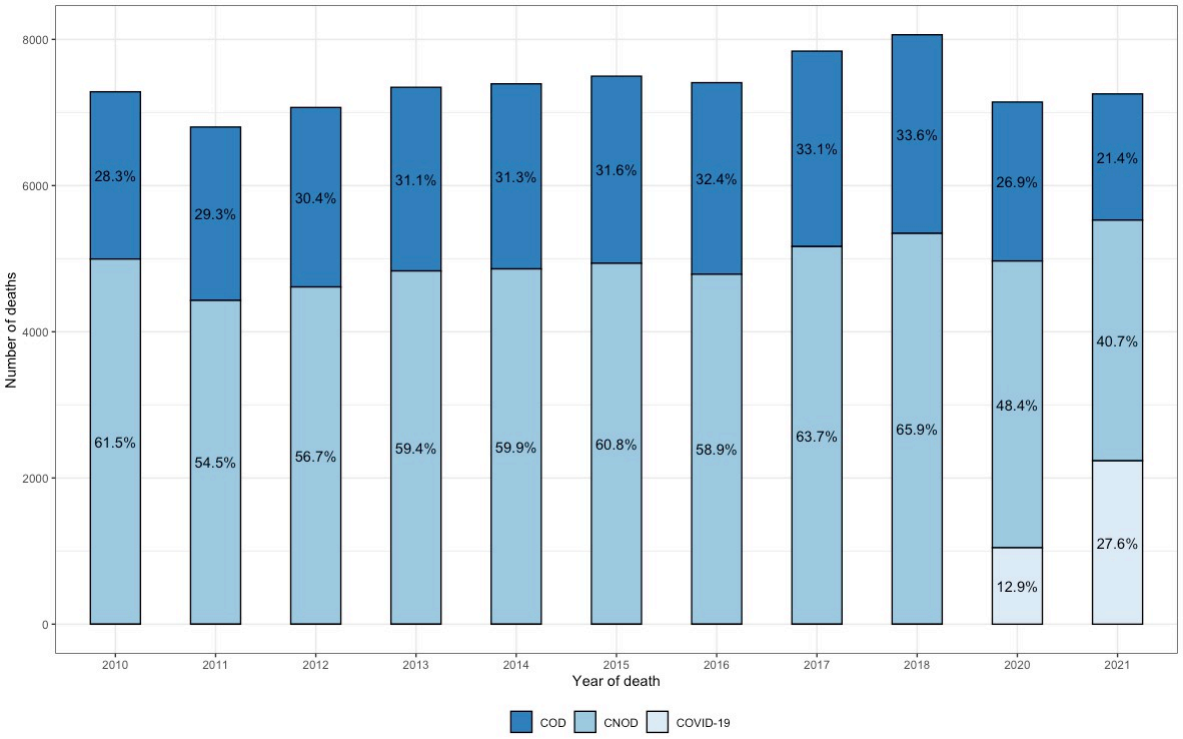
Deaths from diseases with potential need for palliative care according to ICD-10 during 2010–2021 in the Biobío Region, Chile. Percentage of deaths (%). COD: corresponds to the percentage of deaths due to chronic oncological diseases. CNOD: corresponds to the percentage of deaths due to chronic nononcological diseases. COVID-19: represents the percentage of deaths from COVID-19.

Fig 2 shows the number of deaths from all causes from 2010 to 2021 (dark blue line), in addition to those deaths that likely would have required palliative care for COD and CNOD. It is observed that the total deaths in the Biobío Region in the prepandemic period varied from 9,256 deaths in 2010 to 9,921 in 2018, representing an increase of 7.18%, while in the pandemic period (2020–2021), it was reduced to 8,737 deaths (decrease of 11.93% compared to 2018). Considering the sum of deaths from COD and CNOD, Fig 2 shows that, in the prepandemic period (2010–2018), the number of deaths that would have required palliative care increased from 7,261 deaths in 2010 to 8,046 in 2018, representing an increase of 10.81%, of which 65.9% corresponded to CNOD and 33.60% corresponded to COD (Fig 3). On the other hand, in the pandemic period, these deaths decreased to 5,018 in 2021, representing a decrease of 37.76% compared to 2018.

**Fig 4:**
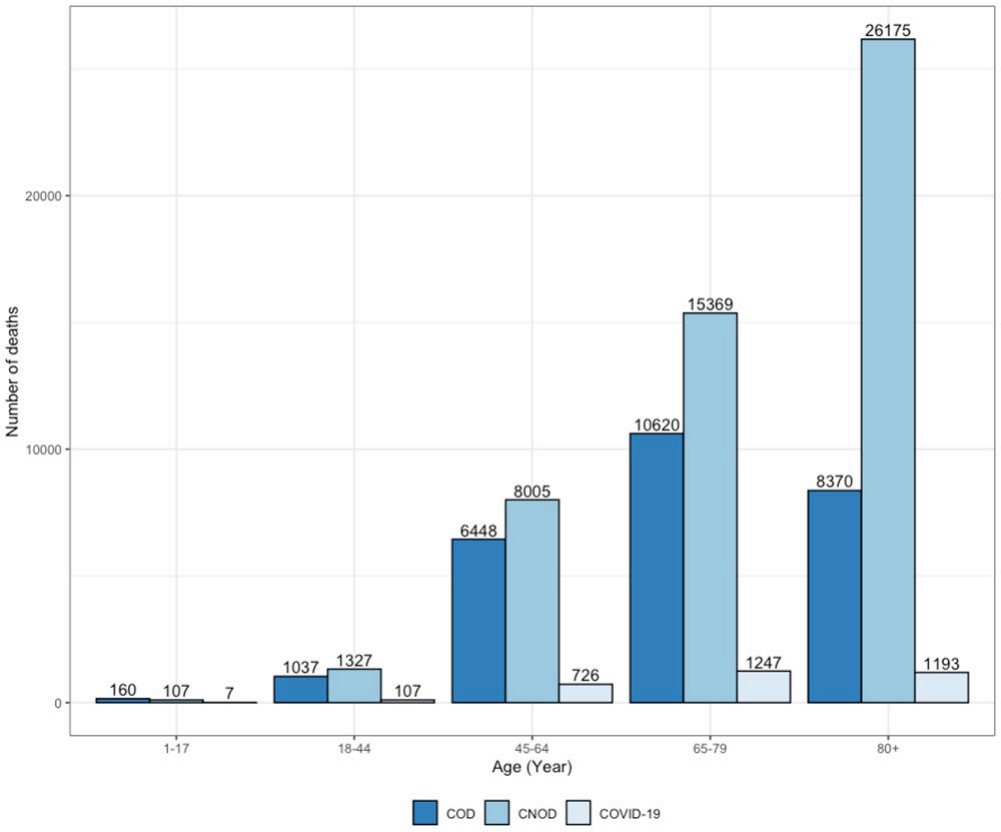
Number of deaths by age groups and causes from 2010 to 2021 in the Biobío Region, Chile. COD: corresponds to the number of deaths from chronic oncological diseases according to age groups. CNOD: corresponds to the number of deaths from chronic nononcological diseases according to age groups. COVID-19: represents the number of deaths from COVID-19 according to age groups.

If the sum of deaths due to CNOD and COD was considered during the entire study period (2010–2021), it is estimated from the mortality records that 77,618 people would have required palliative care. This shows a need for palliative care in 50,983 deaths from CNOD, which represents 50.09% of deaths from all causes in the study period. Similarly, there is evidence of a need for palliative care in 26,635 deaths from COD, which represents 26.17%. Another relevant aspect of the total deaths from CNOD was that 81.49% corresponded to people over 65 years of age, see Fig 4.

Table 2 reports the results obtained by applying the mean-variance tests. We can determine that the average number of CNOD deaths per community after the COVID-19 pandemic was significantly lower than before the pandemic, while the average number of COD deaths per community also decreased during the pandemic period; however, it was not significant at the 5% confidence level, but it was at a 10% confidence level. That is, the difference in the average number of COD deaths per commune was statistically weak. From the above, it can be inferred that people with CNOD during the pandemic period were more likely to die from COVID-19 than from their baseline disease. This difference will be explained below through an analysis of the standardized mortality rates of CNOD and COD by communities during the entire period under study.

**Table 2:** Mean-variance tests. This table reports the results of the mean-variance test of the number of deaths due to COD and CNOD prior and posterior to the pandemic. The symbols ***, **, and * represent significance levels of 1%, 5%, and 10%, respectively.

	Pre-Pandemic			Pandemic			t-welch
	obs	mean	variance	obs	mean	variance	${\bar{X}}_{pre}-{\bar{X}}_{post}$
CNOD	297	147.40	28829.55	66	109.18	15672.67	−38.22**
COD	297	76.54	8260.65	66	59.12	4712.11	−17.42*

### Geographic patterns in the age-standardized mortality rates from COD and CNOD

The geographic patterns of COD and CNOD mortality by commune were analyzed throughout 2010–2021. For this, the recommendations of various authors on the advantages of comparing standardized mortality rates [23, 24] were followed. Specifically, ASDR was used between the prepandemic and pandemic periods.

**Fig 5:**
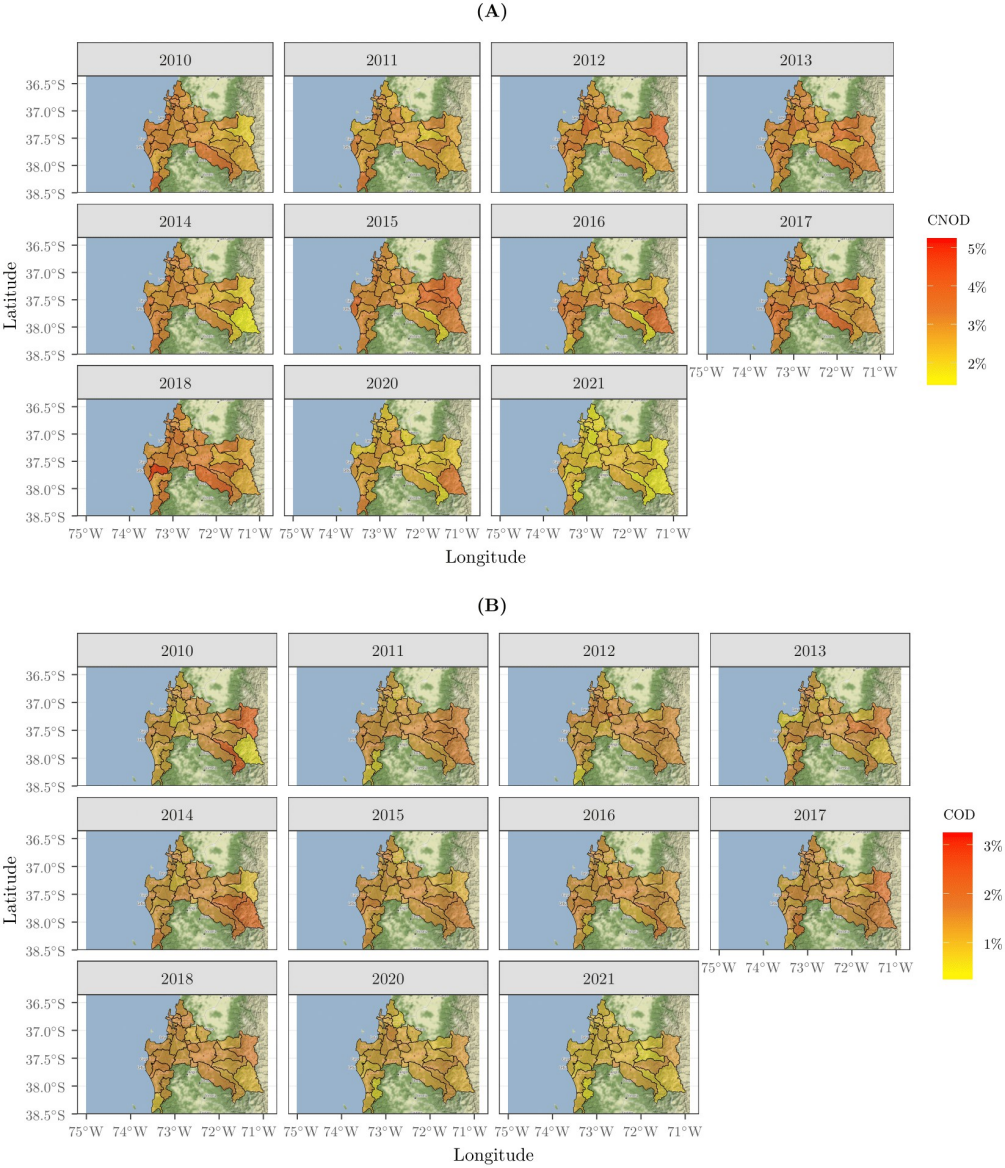
Age-standardized mortality rates during 2010–2021 in the Biobío Region (per 1000). **(A)** Age-standardized mortality rate for CNOD in each community. **(B)** Age-standardized mortality rate for COD in each community. Base map and data from OpenStreetMap and OpenStreetMap Foundation under the Open Database License.

Fig 5 shows the ASDR of COD and CNOD in the Biobío Region during 2010–2021. Panel (a) shows that the CNOD ASDR trend was similar in the prepandemic period, with higher rates in communes in the south of the Biobío Region (Cañete, Contulmo, Los Alamos and Tirúa), southwest (Mulchén, Negrete and Nacimiento), northeast (Antuco, Tucapel, Alto Biobío and Quilleco) and northwest (Talcahuano and Tomé). In addition, it is observed that during the pandemic, the ASDR had a considerable decrease in all communes, with a higher rate in the Tirúa commune. Panel (b) shows the ASDR by COD, where the highest mortality rates were found in the western Biobío Region (Antuco, Quilaco, Alto Biobío, Santa Barbara and Quilleco). On the other hand, the magnitude of the rates in the prepandemic and pandemic periods seems to be constant, since only slight changes were detected in 2021. Finally, it is observed that the ASDR of CNOD had a greater magnitude than the ASDR of COD.

### Effect of the COVID-19 pandemic on the ASDRs of CNOD and COD

The effect of COVID-19 on ASDRs obtained during the pandemic period was analyzed. For this purpose, the model defined in Eq (1) was fitted. Table 3 shows the estimates and goodness-of-fit criteria for both the global OLS method and the estimates obtained from the GWR model.

**Table 3:** Estimated ordinary least squares (OLS) model (global model) and geographically weighted regression (GWR) model (local model). The OLS model explained about 28% of the variance in average ASDR, while the GWR model explained about 38% for CNOD and 27% for COD.

CNOD	OLS Model (Global Model)			GWR Model (Local Model)		
Variable	*β*	*se*(*β*)	t-value	[Min,Max]	[1st Qu, 3rd Qu]	Median
Intercept	0.51	0.64	0.80	[-0.51, 1.18]	[-0.06, 0.77]	0.67
${\bar{ASDR}}^{*}\left(i\right)$	0.52	0.22	2.35*	[0.15, 0.85]	[0.36, 0.67]	0.42
${\bar{COVID}}^{*}\left(i\right)$	0.20	0.16	1.25	[0.19, 0.76]	[0.31, 0.76]	0.35
Adjusted *R*^2^	0.28			0.38		
AICc	28.44			25.32		
Moran’s *I*^2^	27					
**COD**						
Intercept	0.66	0.49	1.34	[-0.83, 2.84]	[0.37, 1.47]	1.30
${\bar{ASDR}}^{*}\left(i\right)$	0.37	0.28	1.31	[-1.01, 1.35]	[-0.01, 0.48]	0.02
${\bar{COVID}}^{*}\left(i\right)$	-0.02	0.10	-0.19	[-0.25, 0.21]	[-0.11, 0.11]	-0.04
Adjusted *R*^2^	-0.01			0.27		
AICc	11.47			9.69		
Moran’s *I*^2^	27					

*p < 0.05. The minimum (Min), maximum (Max), 1st quartile (1st Qu), 3rd quartile (3rd Qu) and median, of GWR coefficient estimates.

Regarding the impact of COVID-19 on the average CNOD ASDR in the pandemic period, this was positive with a global estimated coefficient ${\hat{\beta }}_{2}=0.2$; that is, the average CNOD ASDR increased by 20% for each percentage increase in the average number of deaths from COVID-19. This finding indicated that communes with higher mortality from COVID-19 were more likely to experience deaths from CNOD. Similarly, communes with the highest COVID-19 mortality experienced a 2% ASDR decrease in COD deaths.

The comparison of the *β* values between the dependent variables indicated that the historical ASDR of CNOD was the only significant variable to explain the variation in the average ASDR.

Observing the *R*^2^ adjusted of both methods, it can be noted that the estimated global model (OLS model) explained approximately 28% and the GWR model approximately 38% of the variation of the average ASDR for CNOD, while the OLS explained less than 1% compared to 27% of the GWR model of the variation of the average of the ASDR for COD. This indicates a better performance of the GWR model in explaining the variance of the ASDR average compared to the OLS model.

For both CNOD and COD cases, the lower AICc value of the GWR model (AICc = 25.32; 9.69) indicated a closer approximation of the model to the real nature of the relationship between the average mortality from COVID-19 in each commune and the average ASDR achieved by the OLS model (AICc = 28.44; 11.47).

The two previous criteria indicated that the estimated GWR model better explains the variation in the average ASDR than the estimated OLS model. This suggests that there could be nonstationary effects between the COVID-19 death rates and the COD and CNOD mortality rates during the pandemic period.

**Fig 6:**
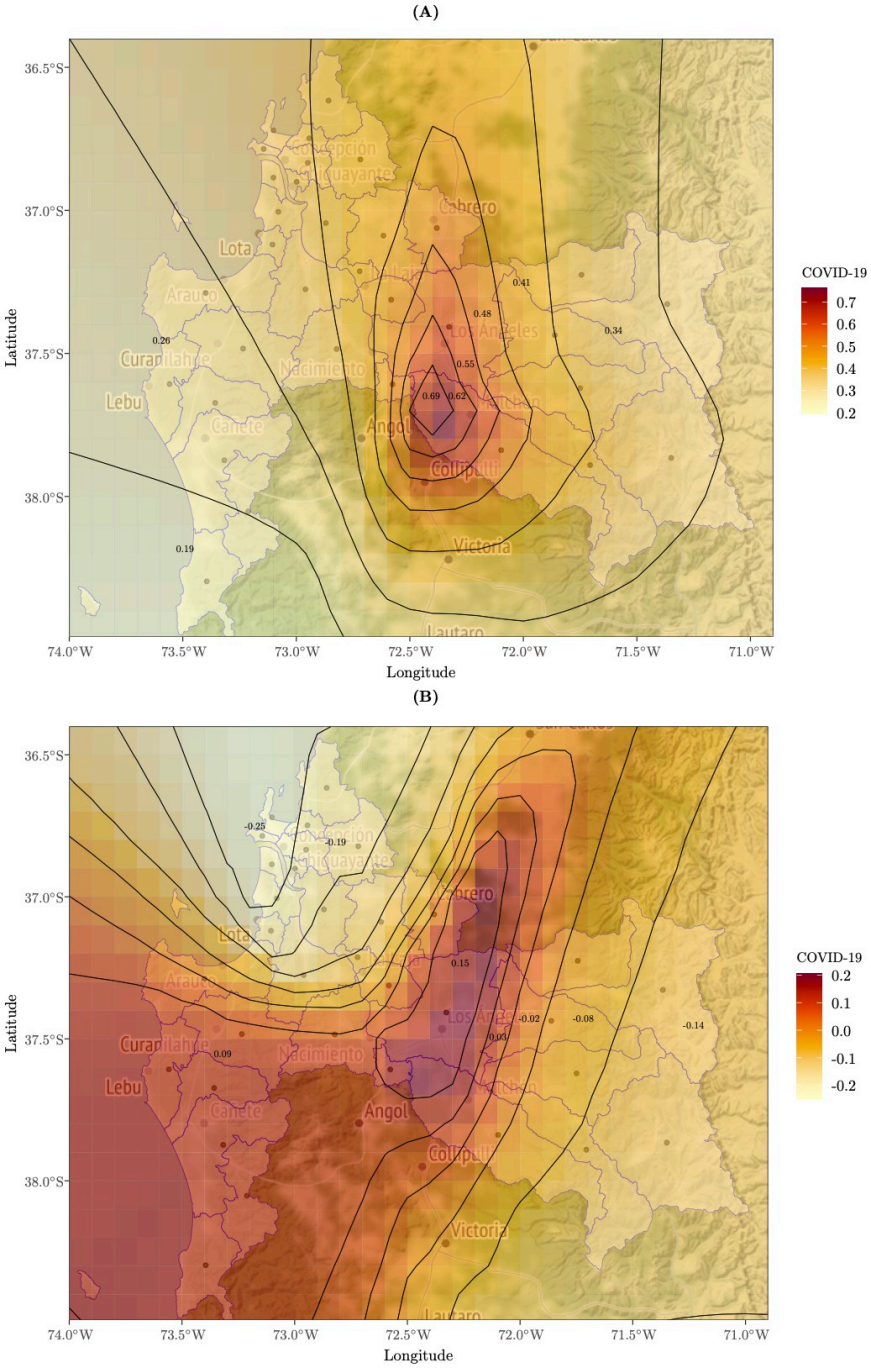
Spatial distribution of geographically weighted regression (GWR) coefficient estimates for COVID-19: **(A)** Local model estimates for the variable *β*_2_(*u*_*i*_, *v*_*i*_) in model (1) at each location *i* to explain age-standardized mortality rates (ASDR) of CNOD.**(B)** Local model estimates of the GWR coefficient for ASDR of COD. Base map and data from OpenStreetMap and OpenStreetMap Foundation under the Open Database License.

Fig 6 shows the spatial heterogeneity of the average ASDR explained by the average COVID-19 death rate in each community. In particular, Panel (a) shows that in the central areas of the region (Los Angeles, Mulchén, Yumbel, Cabrero, Laja, San Rosendo, Negrete and Nacimiento), the estimated coefficient for ${\bar{COVID}}^{*}\left(i\right)$ contributes to a high percentage (60%-70%) that described CNOD’s average ASDR (its estimated global regression coefficient is 20%). In the extreme south of the Biobío Region (Cañete, Tirúa, Contulmo and Lebu), the effect of the average mortality from COVID-19 on the increase in the ASDR of CNOD was weak (19%-26%).

On the other hand, the effect of COVID-19 on the ASDR average of COD is visualized in Panel (b), where it is observed that in the northwest of the Biobío Region, deaths from COVID-19 have a significantly greater impact on the decrease in COD deaths (25%-18%). In this case, the estimated global coefficient is −2%. The same effect, but more attenuated, is seen in the northwestern areas of the Biobío Region (Quilaco, Quileco, Alto Biobío, Antuco, Santa Bárbara, and Tucapel) with a variation of −0.02 to −0.14 (−2% to −14%). In addition, it is observed that in the central (Los Angeles) and southeastern areas of the region (Arauco, Curanilahue, Lebu, Los Alamos, Cañete, Tirúa and Contulmo), the impact of deaths from COVID-19 on deaths from COD was positive, and the magnitude ranged from 10% to 20%; that is, as deaths from COVID-19 increased, deaths from COD increased by between 10% and 20%.

## Discussion

It can be concluded that during the study period, 76.25% of deaths from CNOD and COD in the Biobío Region would have required palliative care, which represents 77,618 people who should have been included in health benefits and palliative care. Of this group, 26.17% were deaths from COD that were probably incorporated into the National Cancer Pain Relief and Palliative Care Program. However, 50.09% were deaths from CNOD, which represents 50,983 people in the Biobío Region who were excluded from palliative care; in this group, 81.49% corresponded to people over 65 years of age.

In Chile, the Cancer Pain Relief and Palliative Care Program currently has an annual coverage of more than 40,000 patients, of which 88% are people with advanced-stage cancer, and 8% are undergoing nonprogressive cancer pain management. With the implementation of Law 21375, it is estimated that it will benefit between 11,000 and 13,000 patients per year, with a projection of national coverage in 2026 of greater than 60,000 people with CNOD and COD [33]. The figure for this projection is below what this study estimated because if only the Biobío Region is considered (77,618 people requiring palliative care), the need at the national level would be much greater.

When considering the prepandemic and pandemic periods, a significant impact was observed in the average number of deaths from CNOD, where people belonging to this group were more likely to die from COVID-19 than from their baseline disease. This is relevant given that, if they had been in control and included in a palliative care program, they could have better coped with the impact of the pandemic. However, considering only the pandemic period, COVID-19 affected the average CNOD ASDR by 20%.

In this line, [15] pointed out that variations between mortality rates in pre- and postpandemic periods could be explained by greater susceptibility to COVID-19 in patients with underlying chronic diseases. In particular, these authors considered a sample of 355 patients who died from COVID-19. If the presence or absence of underlying diseases in these deceased people was considered, only 3 patients (0.8%) did not have underlying diseases, 89 (25.1%) had a single underlying disease, 91(25.6%) had 2 underlying diseases and 172 (48.5%) had 3 or more underlying diseases. In this sample, 117 patients (30%) had ischemic heart disease, 126 (35.5%) had diabetes, 72 (20.3%) had active cancer, 87 (24.5%) had atrial fibrillation, 24 (6.8%) had dementia and 34 (9.6%) had a history of stroke. The diseases described and identified in this study were those with a potential need for palliative care, as indicated in Table 1.

In Chile, [16] evidenced a reduction in the number of deaths from respiratory diseases (DRD) different from COVID-19 during the pandemic period. The above could be explained by the health measures applied by the Ministry of Health of Chile in relation to mobility restrictions and social distancing, among others. These authors point out that a percentage increase in COVID-19 deaths would result in a decrease of 5.987% in the DRD number. These findings confirm the hypothesis of a study conducted in Brazil by [34], in which the authors estimated an average subregistration of 40.68% (range 25.9%-62.7%) for deaths related to COVID-19 in the country. Given the above, and what is evidenced in this study, it can be inferred that the decrease in the mortality rates of CNOD and COD corresponds to an overregistration of mortality by COVID-19. Therefore, it is necessary to establish precise and internationally validated protocols of what is or is not a death related to COVID-19. In the national context, in the data delivered by the Ministry of Health of Chile, the mortality records only indicate the final cause of death and do not declare whether the patient had underlying diseases or comorbidities. Therefore, it is necessary for research contexts to have access to broader information.

In relation to the geographic behavior of the average CNOD ASDR, it is evident that in the central areas of the Biobío Region (Los Angeles, Mulchén, Yumbel, Cabrero, Laja, San Rosendo, Negrete and Nacimiento), the effect of COVID-19 contributed to a significant increase of 60%-70% in CNOD mortality, which raises the need to inquire about health care coverage and the biosociodemographic characteristics of the population living in these areas to better provide for their health care needs.

Regarding the geographic behavior of the average COD ASDR, in the northeast area of the Biobío Region (Quilaco, Quileco, Alto Biobío, Antuco, Santa Bárbara, and Tucapel), the effect of COVID-19 was negative between 5% and 10%, which means a higher probability of death from COVID-19 than from COD in these communes. There is international evidence from Mehta et al. [35] that, with the arrival of the COVID-19 pandemic, palliative care programs faced a great challenge to continue providing care without generating greater risk to patients who were already vulnerable at baseline. To do this, they used telemedicine, video calls and sending prescriptions as a strategy to continue the treatments, which allowed the health team to visualize the home environment of the patients. Notably, there was also a greater proactivity of the patients in terms of planning their care in advance. Ankuda Claire et al. [36] designed an online help program known as the 24–7 PAlliaTive Care Help line (PATCH-24), which provided a guiding light to improve patient care and support clinicians, with 873 of the most difficult cases over 4 weeks during the peak of the COVID-19 pandemic in New York City. Van Houtven et al. [17] noted that approximately 13 million Americans required long-term health care, with timely care delivery difficult due in part to a disparate, fractured system of care and perennial systemic challenges that have been exacerbated by the impact that COVID-19 has generated on organizations and health facilities.

In Chile, Fernández et al. [18] indicated that the country has a solid and efficient national program for people with oncological diseases. In addition, Law 21375 was recently promulgated, which opens palliative care to people with nononcological diseases; however, there are still no clear and definitive guidelines for its implementation. The arrival of the pandemic in Chile oriented efforts early to care for people infected with COVID-19, increasing the capacity of beds in the care network, thus enabling sanitary residences throughout the country and establishing sanitary measures such as quarantines, social isolation, and border closures, among others. This decreased the attendance of patients to palliative care units due to difficulty moving and fear of contagion. In this context, some hospitals incipiently began to provide care remotely by telephone or video calls in coordination with the home hospitalization teams and primary health care centers.

The aforementioned evidence highlights the pressing necessity to commence the implementation of Law 21375, which mandates the provision of palliative care and safeguards the rights of individuals with COD and CNOD. The existing data between 2015 and 2019 indicated in the report by Juan de Dios Parra [37] showed that, in the Biobío Region, the 4 Health Services of the Assistance Network (Concepción, Talcahuano, Biobío and Arauco) provided comprehensive treatment and palliative care for advanced cancer to 69,099 people; meanwhile, they provided comprehensive treatment for pain relief without progressive cancer to 281 people. For a detailed description of palliative care services in the region, we refer readers to the in S1 Table. The Chilean Ministry of Health [38] announced that the economic costs associated with comprehensive treatment and palliative care for advanced cancer have a monthly fee of USD 121.19 and a copayment or out-of-pocket expense of 20% (USD 24.24). The monthly fee for comprehensive treatment for pain relief without progressive cancer is USD 50.28 with a copayment of 20%, which is equivalent to USD 10.06 (calculated with currency values between the Chilean peso and the US dollar, on January 2, 2023). Meanwhile, for people with nononcological diseases, the estimated monthly treatment fee is USD 166.67 with a copayment of USD 33.33, as indicated by the Chilean Chamber of Senators [39] (For more information, see S2 Table). Considering the previous tariffs, it is difficult to implement Law 21375 and provide comprehensive coverage for the entire population that requires it due to the global economic recession caused by the COVID-19 pandemic and the recent war between Russia and Ukraine. However, Chile has guaranteed an initial budget for 2021 for palliative care equivalent to 13 million dollars for nononcological diseases, which will help both the attention of 2,500 people and the hiring and formation of human resources that must operate in primary and secondary care (Medicine, Nursing, Psychology, Kinesiology, Nutrition, among others) [39]. Although these resources will help the initial implementation of a home care model, for the people who had required palliative care only in the Biobio Region, the health network will have required approximately the full budget of 2021 (approximately 12 million dollars). On the other hand, it is important to highlight that palliative care services can be provided at different levels according to the complexity of the needs of patients. At less complex levels, community support networks such as volunteers and other non-governmental organizations can be integrated [40]. The above data lead us to reflect on the need to compromise all public and private entities with the effective application of the new law, since it is currently impossible to achieve the objective of timely palliative care for each person who requires such services. It is noteworthy to mention that this study fulfills the criteria of both population and temporal external validity, as the employed methodology can be applied to a broader range of regions within the country and utilized during various time periods.

Finally, one limitation of this study is the unavailability of sociodemographic variables with relevant characteristics that could contribute to the analysis of COD, CNOD, and social determinants of health (SDH). Variables such as race/ethnicity, socioeconomic level, and housing status, are recognized influencers of the mortality rates among the studied disease groups. Additionally, detailed underlying diagnoses for mortality, other than the primary cause of death attributed to COVID-19, were not accessible for analysis. Consequently, future research could benefit from incorporating not only geographic disparities that impact COD and CNOD mortality but also considering the SDH and the underlying diagnoses associated with the primary cause of death.

## Supporting information

S1 Table Palliative care in the oncology network of the public health system, Biobío Region, Chile. (DOCX)

S2 Table Estimated monthly costs (US$) of palliative care for Cancer and non-cancer patients. (DOCX)
