# Correction: Indirect estimation of the need for palliative care during the COVID-19 pandemic: A descriptive cross-sectional study using mortality data in the Biobío Region, Chile

**DOI:** 10.1371/journal.pone.0319696

**Published:** 2025-02-20

**Authors:** Claudia Barría-Sandoval, Maritza Espinoza Venegas, Guillermo Ferreira

There are errors in the author affiliations. The correct affiliations are as follows:

Claudia Barría-Sandoval^1,2,5*^, Maritza Espinoza Venegas^3^, Guillermo Ferreira^4,5^

**1.** Facultad de Ciencias para el Cuidado de la Salud, Universidad San Sebastián, Sede Concepción, Chile, **2.** Doctorate Program in Nursing, Faculty of Nursing, Universidad de Concepción, Concepción, Chile, **3.** Faculty of Nursing, Universidad de Concepción, Concepción, Chile, **4.** Department of Statistics, Universidad de Concepción, Concepción, Chile, **5.** Grupo de Investigación en el Cuidado Integral de la Comunidad (GICIC), Facultad de Ciencias de Salud, Universidad de las Américas, Chile

The following information is missing from the Funding statement: This publication was supported by the Open Access Support Fund of the Vice-Rector’s Office for Research and Doctorates of the Universidad San Sebastián, Chile. VRID_APC23/12.
