# Supplementary material for: Indirect estimation of the need for palliative care during the COVID-19 pandemic: A descriptive cross-sectional study using mortality data in the Biobío Region, Chile
Source: PLoS One. 2023 Jul 7;18(7):e0288020. doi: 10.1371/journal.pone.0288020 (PMC10328371; doi:10.1371/journal.pone.0288020)
Supplement: S1 Table — (DOCX) [file pone.0288020.s001.docx]

**Table S1. Palliative Care in the Oncology Network of the Public Health System, Biobío Region, Chile.**

| **CONCEPCIÓN** | | | |
| --- | --- | --- | --- |
| **Health Service** | **Type of Health Establishment** | **Commune** | **Palliative Care Development Level** |
| Regional Clinic Dr. Guillermo Grant Benavente | Hospital | Concepción | 3 |
| San José | Hospital | Coronel | 3 |
| Lota Hospital | Hospital | Lota | 3 |
| Clorinda Avello Hospital | Hospital | Santa Juana | 3 |
| San Agustín Hospital | Hospital | Florida | 3 |
| Victor Manuel Fernández | Family Health Center | Concepción | 3 |
| Tucapel | Family Health Center | Concepción | 3 |
| Juan Soto Fernández | Family Health Center | Concepción | 3 |
| Lorenzo Arenas | Family Health Center | Concepción | 3 |
| Santa Sabina | Family Health Center | Concepción | 3 |
| Pedro de Valdivia | Family Health Center | Concepción | 3 |
| O’Higgins | Family Health Center | Concepción | 3 |
| Villa Nonguén | Family Health Center | Concepción | 3 |
| Boca Sur | Family Health Center | San Pedro | 3 |
| San Pedro | Family Health Center | San Pedro | 3 |
| San Pedro de la Costa | Family Health Center | San Pedro | 3 |
| Loma Colorada | Family Health Center | San Pedro | 3 |
| Leonera | Family Health Center | Chiguayante | 3 |
| Chiguayante | Family Health Center | Chiguayante | 3 |
| Pinares | Family Health Center | Chiguayante | 3 |
| Yobilo | Family Health Center | Coronel | 3 |
| Lagunillas | Family Health Center | Coronel | 3 |
| Carlos Pinto Fierro | Family Health Center | Coronel | 3 |
| Dr. Juan Cartes Arias | Family Health Center | Lota | 3 |
| Dr. Sergio Lagos Olave | Family Health Center | Lota | 3 |
| Hualqui | Family Health Center | Hualqui | 3 |
| TALCAHUANO | | | |
| **Health Service** | **Type of Health Establishment** | **Commune** | **Palliative Care Development Level** |
| The Higueras | Hospital | Talcahuano | 2 |
| Penco-Lirquén | Hospital | Penco | 2 |
| Tomé | Hospital | Tomé | 2 |
| **BiOBÍO** | | | |
| **Health Service** | **Type of Health Establishment** | **Commune** | **Palliative Care Development Level** |
| Dr. Victor Ríos Ruiz Assistance Complex | Hospital | Los Ángeles | 1 |
| **ARAUCO** | | | |
| **Health Service** | **Type of Health Establishment** | **Commune** | **Palliative Care Development Level** |
| San Vicente | Hospital | Arauco | 2 |
| Dr. Hans Gronemann | Hospital | Contulmo | 2 |
| Kallvu Llanka Intercultural | Hospital | Cañete | 2 |
| Saint Elizabeth of Lebu | Hospital | Lebu | 2 |
| Provincial Dr. Rafael Avaria Valenzuela | Hospital | Curanilahue | 2 |
| **Palliative Care Development Level** | | | |
| Palliative Care | 1 | Health Services that perform Palliative Care only in High Complexity Hospitals. | |
|  | 2 | Health Services that perform Palliative Care only in High, Medium and Low Complexity Hospitals. | |
|  | 3 | Health Services that perform Palliative Care only in Hospitals (High, Medium and Low Complexity), and in Family Health Center (Primary Care). | |

^Source:: Ministry of Health of Chile: National Cancer Plan 2018-2028:^ [^https://cdn.digital.gob.cl/filer_public/d3/0a/d30a1f5e-53d9-4a31-a4fe-e90d8d9a2348/documento_plan_nacional_de_cancer.pdf^.](https://cdn.digital.gob.cl/filer_public/d3/0a/d30a1f5e-53d9-4a31-a4fe-e90d8d9a2348/documento_plan_nacional_de_cancer.pdf)
