# Supplementary material for: Indirect estimation of the need for palliative care during the COVID-19 pandemic: A descriptive cross-sectional study using mortality data in the Biobío Region, Chile
Source: PLoS One. 2023 Jul 7;18(7):e0288020. doi: 10.1371/journal.pone.0288020 (PMC10328371; doi:10.1371/journal.pone.0288020)
Supplement: S2 Table — (DOCX) [file pone.0288020.s002.docx]

**S2 Table. Estimated Monthly Costs (US$) of Palliative Care for Cancer and Non-Cancer Patients.**

| **Economic costs of treatment for pain management and palliative care in people with cancer ^(a)^** | | | | |
| --- | --- | --- | --- | --- |
| **Type of Health Care** | **Benefit or Group of Benefits** | **Periodicity** | **Tariff** | **Copay 20%** |
| Treatment | Comprehensive treatment and palliative care for advanced cancer | Monthly | USD 121.19 | USD24.24 |
|  | Comprehensive treatment for pain relief without progressive cancer | Monthly | USD 50.28 | USD 10.06 |
| **Economic costs of treatment for pain management and palliative care in people with non-cancer ^(b)^** | | | | |
| Treatment | Comprehensive treatment and non-oncological palliative care | Monthly | USD 166.67 | USD 33.33 |

Source note: ^(a)^Minister of Health (2019): <https://auge.minsal.cl/problemasdesalud/index/4>. ^(b)^Senate of Chile <https://www.senado.cl/noticias/eutanasia/cuidados-paliativos-norma-pasa-a-la-comision-de-hacienda-para-revisar>.
